# Related factors affecting misdiagnosis of aortic dissection: a single-center retrospective study

**DOI:** 10.3389/fcvm.2025.1561225

**Published:** 2025-04-14

**Authors:** Sheng Wang, Liu Yang, Tao Hu, Hui Deng, Weiling Tu, Yijie Wu, Linfeng Li

**Affiliations:** ^1^ Jiangxi Medical College, Nanchang University, Nanchang, Jiangxi, China; ^2^ Jiangxi Provincial People’s Hospital, The First Affiliated Hospital of Nanchang Medical College, Nanchang, Jiangxi, China

**Keywords:** aortic dissection, diagnosis challenge, misdiagnosis, predictive modeling, risk factors aortic dissection, risk factors

## Abstract

**Objective:**

Aortic dissection (AD) is a life-threatening cardiovascular emergency. Delayed diagnosis frequently leads to treatment delays, elevated mortality, and complications. This study investigates the factors contributing to the misdiagnosis of AD and proposes strategies for improving its early diagnosis.

**Methods:**

A retrospective analysis of 801 patients with AD identified 219 cases for inclusion, which were split into a training set (131 cases) and a validation set (88 cases). A binary logistic regression model was used to identify factors influencing misdiagnosis, while a Nomogram prediction model was developed.

**Results:**

The analysis revealed that factors such as the timing and suddenness of symptom onset, typical back pain, walk-in clinic visits, and laboratory results (D-dimer, fibrinogen, and white blood count) were significant in predicting misdiagnosis. The Nomogram model showed high predictive accuracy with an Area under the ROC curve (AUC) of 0.924 in the training set and 0.912 in the validation set, demonstrating good sensitivity and specificity.

**Conclusion:**

The model offers potential for improving diagnostic accuracy and clinical outcomes in AD cases.

## Introduction

Aortic Dissection (AD) is a catastrophic cardiovascular disease. According to reports (1), the incidence of AD in recent years is 4.8 per 100,000 person-years, and the mortality rate increased by 1%–3% per hour without treatment, reaching up to 25% within 24 h (2). Therefore, timely and accurate diagnosis and treatment are crucial. However, the complex and variable clinical manifestations of AD often lead to misdiagnosis. Some reports (3) have found that the misdiagnosis rate of AD ranges from 14% to 78%, with atypical symptoms being the most critical factor for misdiagnosis. AD's clinical heterogeneity complicates diagnosis. Even patients with classic symptoms (e.g., sudden chest/back pain) are often misdiagnosed as acute coronary syndrome (ACS) or pulmonary embolism (PE) due to overlapping presentations (4, 5). Once misdiagnosed, it not only prolongs the treatment time for AD but also increases the risk of coagulation dysfunction and perioperative bleeding due to routine preoperative anticoagulation or antiplatelet therapy, making the decision for emergency surgery difficult (6). Furthermore, approximately 6% of patients with aortic dissection (AD) present without pain, instead manifesting as syncope, stroke, or heart failure—scenarios that significantly increase the risk of misdiagnosis (7). While aortic computed tomographic angiography (CTA) is considered the gold standard for diagnosing AD, its high cost and the use of iodine contrast agents pose risks, particularly acute renal impairment in patients with pre-existing renal dysfunction. Consequently, CTA may be overlooked in cases with atypical clinical presentations or inadequate risk assessment by the attending physician, potentially leading to delayed diagnosis. It is important to note that the diagnostic accuracy of CTA is not infallible. For instance, Paulraj reported a case of Stanford type A aortic dissection that was not detected by CTA but was suggested by transthoracic echocardiography (TTE) and ultimately confirmed by transesophageal echocardiography (TEE) (8).Therefore, identifying predictors of misdiagnosis is essential to improve early detection rates and reduce associated mortality. The purpose of this study was to examine and ascertain the factors correlated with the misdiagnosis of AD. Through the examination of patient demographics, clinical presentation, and initial diagnostic approaches, our objective was to investigate potential factors that could contribute to diagnostic inaccuracies. This advancement could facilitate the precise and prompt diagnosis of AD, ultimately enhancing patient outcomes and alleviating the healthcare burden linked to this condition.

## Patients and methods

### Patients

Patients diagnosed with aortic dissection upon discharge from Jiangxi Provincial People's Hospital between January 1, 2017 and December 31, 2023 were gathered retrospectively using electronic medical record system, and their outpatient and hospitalization records were subsequently followed up. We have access to information that could identify individual participants during or after data collection. Inclusion criteria: (1) patients presenting with symptomatic manifestations upon admission; (2) no prior history of aortic dissection; (3) a confirmed diagnosis of aortic dissection established through aortic CTA/Transesophageal echocardiography (TEE)/Magnetic Resonance Imaging (MRI). Exclusion criteria: (1) a confirmed or suspected diagnosis of aortic dissection at an external hospital; (2) a known history of aortic dissection; (3) consideration of aortic dissection based solely on ultrasound or plain CT without confirmation via thoracoabdominal aortic CTA; (4) incomplete clinical data.

### Definition of misdiagnosis

(1) Failed to perform appropriate diagnostic tests. All cases of suspected AD should have the presence of intimal tear, true or false lumen, and branch vessel involvement clarified by CTA. If the patient is unable to undergo immediate CTA due to renal insufficiency, contrast allergy, or emergency conditions, alternative confirmation is required by one of the following tests. TEE: should be performed by an experienced sonographer to clearly show signs of aortic intimal tear or coarctation.MRI: for stabilized patients and needs to be performed after prioritizing the exclusion of contraindications in emergency conditions.
(2) AD was excluded from all initial admission diagnoses and differential diagnoses.Two independent cardiovascular physicians blindly reviewed the patient's initial admission record (including chief complaint, physical examination, initial diagnosis and differential diagnosis list) through the electronic medical record system.

If AD was not mentioned as a differential diagnosis in the medical record, or if no specific tests (e.g., D-dimer test, imaging) were performed for AD, it was determined to be a “misdiagnosis”.

For cases where CTA cannot be completed immediately due to critical illness (e.g., shock, impaired consciousness), if AD is included in the differential diagnosis and confirmatory tests are planned after stabilization, such cases are not classified as misdiagnosis. Misdiagnosis was only assigned when AD was entirely excluded and no confirmatory tests were scheduled. All misdiagnosis determinations were based on objective medical records, avoiding subjective speculation. The study team was not involved in clinical decision-making of patients and only performed retrospective data analysis.

The study complied with the 2024 revised Declaration of Helsinki and was approved by the Medical Ethics Committee of Jiangxi Provincial People's Hospital. Given its retrospective nature, the requirement for informed consent was waived.

Ethics Approval Code: Kekuai 2024 (75).

### Clinic baseline

The relevant information was gathered through the electronic patient record system. Basic information: gender, age, mode of admission. Clinical features include heart rate, systolic and diastolic blood pressure, the timing of symptom onset, whether the onset was sudden, the presence and location of pain (e.g., head, neck, chest, back, lumbar region, abdomen), the new onset of a murmur in the aortic valve area, and other symptoms such as dyspnea, profuse sweating, chest tightness, palpitations, cough, fever, neurological deficits, and nausea or vomiting. Past medical history: including hypertension, coronary artery disease, diabetes mellitus, stroke, chronic kidney disease, and a history of smoking. Laboratory tests: D-dimer, fibrinogen levels, and white blood count, platelet count, and troponin. Imaging and other diagnostic tools: electrocardiogram, cardiac and abdominal ultrasound, chest and abdominal CT, thoracic and abdominal aortic CTA. Disease diagnosis and treatment: Aortic dissection staging (Stanford staging), whether or not the patient received surgical treatment. Prognosis and outcome: mortality during hospitalization, initial diagnosis of misdiagnosed patients, and modes of correction.

### Statistical analysis

This study was statistically analyzed using the R language (version 4.4.1). Count data were expressed as mean ± standard deviation (for normal distribution) or median and interquartile range (for non-normal distribution). Differences between groups were analyzed using *t*-test (for normal distribution) or Wilcoxon rank-sum test (for non-normal distribution). Metric data were presented as percentages, and group differences were assessed through chi-square test. The possible factors affecting the misdiagnosis of aortic coarctation were analyzed through one-way analysis. The indicators with *P* < 0.1 were then regressed backward step by step to create a multifactorial logistic regression prediction model. The model was analyzed using the Receiver Operating Characteristic (ROC) curve and the Area Under the Curve (AUC). A calibration curve was also used to assess the model's performance. The model was validated using the ROC curve, AUC, and a Decision Curve Analysis (DCA) to evaluate its usefulness in clinical settings.

## Results

A total of 801 patients were diagnosed with AD upon discharge. After applying the exclusion criteria, 582 cases were excluded, resulting in a final cohort of 219 patients, of whom 133 were in the confirmed diagnosis group and 86 in the misdiagnosis group. Table 1 presents all the clinical data of the patients. Among the 219 patients, 86 were misdiagnosed, resulting in a misdiagnosis rate of 39.3%. The average age of the patients was 59.4 ± 14.7 years, with a male-to-female ratio of 3:1. Hypertension was the most common underlying condition. A majority of the patients (73.1%) were admitted via ambulance, while 26.9% arrived on foot. The majority of symptom onset occurred within one day (57.1%). Pain was the initial symptom in 75.1% of patients, but only 33.3% described the onset as sudden. Among patients reporting pain, chest pain was the most common (51.1%), and the most frequent non-pain symptom was chest tightness (26.9%). The rate of abnormal ECG findings was 62.1%, while the positivity rates for ultrasound and CT were 56.2% and 79.9%, respectively. Among the 219 aortic dissection patients, 90 (41.1%) were classified as type A, and 129 (58.9%) as type B. A total of 116 patients (53.0%) underwent surgical treatment, with 25 patients dying during hospitalization, resulting in a mortality rate of 11.4%. The top five alternative diagnoses among the 86 misdiagnosed patients were suspected coronary artery disease (14 cases), acute coronary syndrome (10 cases), pulmonary infection (7 cases), acute cerebrovascular disease (6 cases), and heart valve disease (5 cases). In 67 cases (78%), the misdiagnosis was corrected through chest/abdominal CT or transthoracic/abdominal ultrasound.

**Table 1:** Clinical information of patients.

Category	Value
Gender, *n* (%)	
Male	164 (74.9%)
Female	55 (25.1%)
Age (years)	59.4 ± 14.7
Heart Rate (beats/min)	80.7 ± 16.2
SBP (mmHg)^a^	145.8 ± 34.2
DBP (mmHg)^b^	82 ± 20.1
Past history	
Hypertension	152 (69.4%)
Coronary Heart Disease	16 (7.3%)
Diabetes	14 (6.3%)
Stroke	12 (5.4%)
Renal Insufficiency	7 (3.1%)
Smoking	53 (24.2%)
Mode of Admission	
Ambulance	160 (73.1%)
Walk-in	59 (26.9%)
Time of Symptom Onset (day)	
<1	125 (57.1%)
<3	27 (12.3%)
<7	21 (9.6%)
≥7	46 (21%)
Sudden Onset of Symptom	73 (33.3%)
Pain	166 (75.1%)
Pain Location	
Head	2 (0.9%)
Neck	2 (0.9%)
Shoulder	6 (2.7%)
Chest	112 (51.1%)
Back	77 (35.1%)
Waist	16 (7.3%)
Abdomen	30 (13.7%)
New Aortic Valve Murmur	19 (8.7%)
Other Symptoms	
Dyspnea	25 (11.4%)
Profuse Sweating	30 (13.7%)
Chest Tightness	59 (26.9%)
Palpitations	4 (1.8%)
Cough	9 (4.1%)
Fever	8 (3.6%)
Neurological Deficits	40 (18.3%)
Nausea and Vomiting	11 (5.0%)
Hematological Tests	
D-Dimer (mg/L)	1.98 (0.76, 5.98)
Fibrinogen (g/L)	2.59 (2.02, 3.65)
WBC (10^9^/L)^c^	10.0 (7.0, 13.39)
PLT (10^9^/L)^d^	173 (140.0, 207.0)
Troponin I Positive (>0.1 ng/L)	55 (25.1%)
Imaging Examinations	
Any ST-T Segment Abnormalities on ECG	136 (62.1%)
Positive Chest/Abdomen Ultrasound^e^	123 (56.2%)
Positive Chest/Abdomen CT	175 (79.9%)
Diagnosis/Misdiagnosis	133/86
Stanford Classification	
Type A	90 (41.1%)
Type B	129 (58.9%)
Surgical Treatment	116 (53.0%)
Death During Hospitalization	25 (11.4%)
Initial Diagnosis of Misdiagnosed Patients	
Suspected Coronary Heart Disease	14 (16.3%)
Acute Coronary Syndrome	10 (11.6%)
Lung Infection	7 (8.1%)
Acute Cerebrovascular Disease	6 (7.0%)
Cardiac Valvular Disease	5 (5.8%)
Correction Method for Misdiagnosed Patients	
Chest/Abdomen CT	51 (59.3%)
Chest/Abdomen Ultrasound	16 (18.6%)
Aortic CTA	11 (12.8%)
Other	8 (9.3%)

^a^ SBP, systolic blood pressure.

^b^ DBP, diastolic blood pressure.

^c^ WBC, white blood cell.

^d^ PLT, platelet.

^e^ Positive Chest/Abdomen Ultrasound and Chest/Abdomen CT: On chest/abdominal CT and chest/abdominal ultrasound, further refinement of aortic CTA was defined as positive if direct signs of aortic dissection (e.g., intima-media sheet, separation of true lumen from false lumen) or indirect signs (e.g., aortic dilatation, false lumen thrombus, branch vessel involvement, pericardial or pleural effusion) were found to require further refinement of the aortic CTA.

The data presented in Table 2 indicates that a total of 219 patients were allocated into a training set and a test set at a ratio of 3:2. Specifically, 131 patients were assigned to the training set while 88 patients were placed in the test set. The comparison of clinical data between the training set and the test set revealed that a history of diabetes mellitus, back pain, and fibrinogen were found to be statistically significant (The *p*-values were 0.09, 0.09, and 0.035), whereas the remaining indicators did not show statistical significance (*P*-values >0.05).

**Table 2:** Comparison of clinical data between training and validation sets.

Category	Training set (*n* = 131)	Test set (*n* = 88)	*P*
Baseline Data			
Gender, *n* (%)			0.546
Male	100 (76.34)	64 (72.73)	
Female	31 (23.66)	24 (27.27)	
Age (years), *n* (%)			0.347
<60	60 (45.80)	46 (52.27)	
≥60	71 (54.20)	42 (47.73)	
Heart Rate	80.98 ± 16.04	80.33 ± 16.52	0.773
SBP	141.87 ± 33.47	150.86 ± 34.83	0.056
DBP	80.49 ± 20.00	84.64 ± 19.98	0.134
Past history, *n* (%)			
Hypertension	90 (68.70)	62 (70.45)	0.783
Coronary Heart Disease	8 (6.11)	8 (9.09)	0.405
Diabetes	13 (9.92)	1 (1.14)	**0**.009*
Stroke	10 (7.63)	2 (2.27)	0.160
Renal Insufficiency	5 (3.82)	2 (2.27)	0.806
Smoking	31 (23.66)	22 (25.00)	0.821
Mode of Admission			0.688
Walk-in	34 (25.95)	25 (28.41)	
Ambulance	97 (74.05)	63 (71.59)	
Time of Symptom Onset (day)			0.485
<1	70 (53.44)	55 (62.50)	
<3	18 (13.74)	9 (10.23)	
<7	12 (9.16)	9 (10.23)	
≥7	31 (23.66)	15 (17.05)	
Sudden Onset of Symptoms, *n* (%)	42 (32.06)	31 (35.23)	0.626
Pain	96 (73.28)	70 (79.55)	0.289
Pain Location			
Head	1 (0.76)	1 (1.14)	1.000
Neck	2 (1.53)	0 (0.00)	0.517
Shoulder	5 (3.82)	1 (1.14)	0.442
Chest	67 (51.15)	45 (51.14)	0.999
Back	37 (28.24)	40 (45.45)	**0**.009*
Waist	10 (7.63)	6 (6.82)	0.820
Abdomen	20 (15.27)	10 (11.36)	0.410
New Aortic Valve Murmur	13 (9.92)	6 (6.82)	0.423
Other Symptoms			
Dyspnea	15 (11.45)	10 (11.36)	0.984
Profuse Sweating	18 (13.74)	12 (13.64)	0.982
Chest Tightness	38 (29.01)	21 (23.86)	0.400
Palpitations	2 (1.53)	2 (2.27)	1.000
Cough	6 (4.58)	3 (3.41)	0.936
Fever	6 (4.58)	2 (2.27)	0.600
Neurological Deficits	25 (19.08)	15 (17.05)	0.702
Nausea and Vomiting	8 (6.11)	3 (3.41)	0.561
Hematological Tests, M (Q_1_, Q_3_)			
D-Dimer (mg/L)	2.29 (0.76, 6.85)	1.64 (0.79, 4.67)	0.389
Fibrinogen (g/L)	2.87 (2.08, 3.89)	2.42 (1.93, 3.18)	**0**.035*
WBC (10^9^/L)	10.22 (6.90, 13.10)	9.79 (7.25, 13.64)	0.886
PLT (10^9^/L)	173.00 (138.50, 206.00)	172.50 (143.25, 207.25)	0.930
Troponin I, *n* (%)			0.504
Negative	96 (73.28)	68 (77.27)	
Positive	35 (26.72)	20 (22.73)	
Imaging Examinations, *n* (%)			
ECG			0.701
Normal	51 (38.93)	32 (36.36)	
Any ST-T Segment Changes	80 (61.07)	56 (63.64)	
Chest/Abdomen Ultrasound			0.341
Negative	54 (41.22)	42 (47.73)	
Positive	77 (58.78)	46 (52.27)	
Chest/Abdomen CT			0.912
Negative	26 (19.85)	18 (20.45)	
Positive	105 (80.15)	70 (79.55)	

*P < 0.05.

When comparing the two groups of patients through univariate analysis, significant differences were found in age, history of coronary artery disease, mode of admission, time of symptom onset, whether the symptom was sudden, pain, chest pain, back pain, profuse sweating, chest tightness, neurological deficits, D-dimer, fibrinogen, and white blood count. However, the other indicators did not show statistical significance, as indicated in Table 3. Indicators that showed statistical significance in the univariate analysis of the training set were included in the multifactorial regression if they had a *P* value of less than 0.1. Subsequently, a backward stepwise regression analysis was conducted, resulting in the inclusion of white blood count, fibrinogen, D-dimer, back pain, sudden onset of symptoms, time of symptom onset, and mode of admission in the final multifactorial analysis.

**Table 3:** Comparison of clinical data in the training Set.

Category	Confirmed (*n* = 77)	Misdiagnosed (*n* = 54)	Univariate	Multivariate	OR (95%CI)^a^
Gender, *n* (%)			0.745		
Male	58 (75.32)	42 (77.78)			
Female	19 (24.68)	12 (22.22)			
Age (years), *n* (%)			0.041*		
<60	41 (53.25)	19 (35.19)			
≥60	36 (46.75)	35 (64.81)			
Heart Rate	79.36 ± 14.98	83.28 ± 17.32	0.170		
SBP	144.62 ± 38.17	137.94 ± 25.14	0.230		
DBP	80.61 ± 22.22	80.31 ± 16.53	0.931		
Past history, *n* (%)					
Hypertension	58 (75.32)	32 (59.26)	0.051		
Coronary Heart Disease	0 (0.00)	8 (14.81)	**0**.002*		
Diabetes	7 (9.09)	6 (11.11)	0.703		
Stroke	4 (5.19)	6 (11.11)	0.357		
Renal Insufficiency	2 (2.60)	3 (5.56)	0.684		
Smoking	19 (24.68)	12 (22.22)	0.745		
Mode of Admission, *n* (%)			**0**.015*	0.147	0.34 (0.08–1.47)
Walk-in	26 (33.77)	8 (14.81)			
Ambulance	51 (66.23)	46 (85.19)			
Time of Symptom Onset (day)			**<**.001*		
<1	59 (76.62)	11 (20.37)			
<3	11 (14.29)	7 (12.96)		0.100	3.46 (0.79–15.15)
<7	3 (3.90)	9 (16.67)		0.105	5.16 (0.71–37.52)
≥7	4 (5.19)	27 (50.00)		**0**.001*	30.27 (3.94–232.63)
Sudden Onset of Symptoms	39 (50.65)	3 (5.56)	**<**.001*	**0**.002*	0.08 (0.02–0.40)
Pain	71 (92.21)	25 (46.30)	**<**.001*		
Pain Location					
Head	1 (1.30)	0 (0.00)	1.000		
Neck	1 (1.30)	1 (1.85)	1.000		
Shoulder	3 (3.90)	2 (3.70)	1.000		
Chest	50 (64.94)	17 (31.48)	**<**.001*		
Back	33 (42.86)	4 (7.41)	**<**.001*	**0**.003*	0.10 (0.02–0.45)
Waist	9 (11.69)	1 (1.85)	0.080		
Abdomen	12 (15.58)	8 (14.81)	0.904		
New Aortic Valve Murmur	8 (10.39)	5 (9.26)	0.831		
Other Symptoms					
Dyspnea	4 (5.19)	11 (20.37)	**0**.007*		
Profuse Sweating	16 (20.78)	2 (3.70)	**0**.005*		
Chest Tightness	19 (24.68)	19 (35.19)	0.192		
Palpitations	1 (1.30)	1 (1.85)	1.000		
Cough	2 (2.60)	4 (7.41)	0.383		
Fever	0 (0.00)	6 (11.11)	**0**.010*		
Neurological Deficits	13 (16.88)	12 (22.22)	0.444		
Nausea and Vomiting	7 (9.09)	1 (1.85)	0.183		
Hematological Tests, M (Q_1_, Q_3_)					
D-Dimer (mg/L)	3.38 (1.38, 9.85)	1.06 (0.32, 3.90)	**<**.001*	**0**.033*	0.94 (0.90–0.99)
Fibrinogen (g/L)	2.52 (1.97, 3.44)	3.19 (2.29, 4.36)	**0**.018*	0.087	0.63 (0.37–1.07)
WBC (10^9^/L)	12.20 (10.10, 14.15)	7.41 (5.79, 9.54)	**<**.**001**	0.070	0.88 (0.77–1.01)
PLT (10^9^/L)	172.00 (133.00, 198.00)	186.00 (146.25, 236.75)	0.059		
Troponin I, *n* (%)			0.567		
Negative	55 (71.43)	41 (75.93)			
Positive	22 (28.57)	13 (24.07)			
Imaging, *n* (%)					
ECG			0.461		
Normal	32 (41.56)	19 (35.19)			
Any ST-T Segment Changes	45 (58.44)	35 (64.81)			
Chest/Abdomen Ultrasound			0.789		
Negative	31 (40.26)	23 (42.59)			
Positive	46 (59.74)	31 (57.41)			
Chest/Abdomen CT			0.227		
Negative	18 (23.38)	8 (14.81)			
Positive	59 (76.62)	46 (85.19)			

^a^ OR, odds ratio, CI, confidence interval.

*P < 0.05.

**Figure 1:**
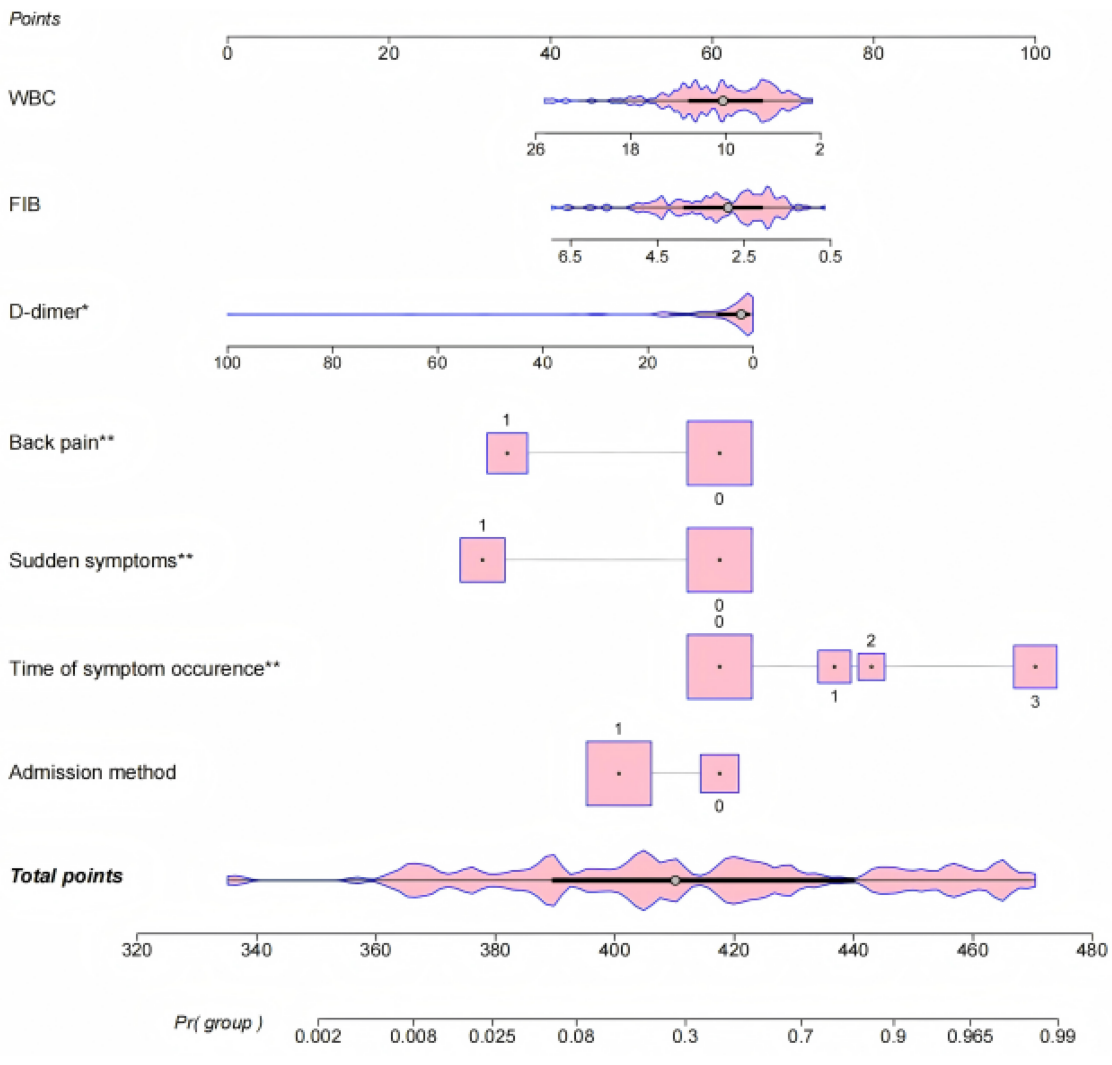
Nomogram model for predicting factors contributing to misdiagnosis of AD.

The results of logistic regression analyses were used to create a nomogram to predict the risk of misdiagnosis of AD based on seven significant correlates. Each categorical variable was assigned a value: back pain (none = 0, yes = 1), sudden onset of symptoms (none = 0, yes = 1), duration of symptoms (<1 day = 0, <3 days = 1, <7 days = 2, ≥7 days = 3), and mode of admission (walk-in = 0, ambulance = 1). The regression coefficients were converted to scores ranging from 0 to 100. The nomogram (Figure 1) shows that for patients with AD, the time of symptom onset was the strongest predictor of misdiagnosis. Longer duration of symptoms increased the likelihood of misdiagnosis. Other predictors of misdiagnosis included non-sudden onset of symptoms, absence of typical back pain, walk-in emergency, and normal or low levels of D-dimer, fibrinogen, and white blood count.

**Figure 2:**
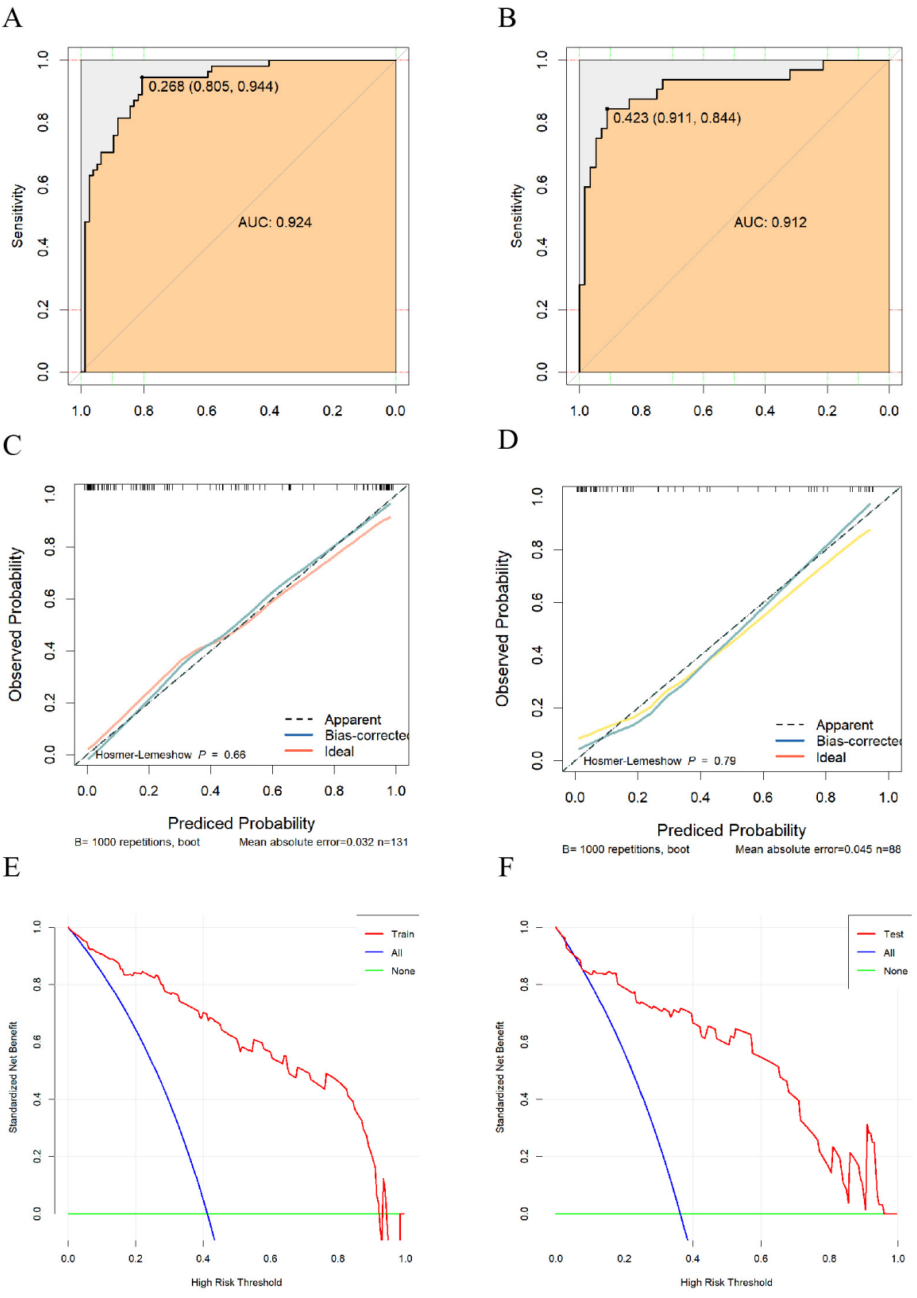
**(A)** ROC curve of the training set; **(B)** ROC curve of the validation set; **(C)** calibration curve of the training set; **(D)** calibration curve of the validation set; **(E)** DCA curve of the training set; **(F)** DCA curve of the validation set.

Validation of the fitting effect and goodness-of-fit of the predictive model for factors affecting misdiagnosis of AD should be conducted. The risk prediction model's discrimination was evaluated by generating ROC curves. In the training set, the AUC was 0.924 (95% CI: 0.879–0.970), with a cutoff value of 0.268, sensitivity of 0.94 (95% CI: 0.883–1.000), and specificity of 0.805 (95% CI: 0.717–0.894) (Figure 2A). Additionally, the validation set showed an AUC of 0.912 (95% CI: 0.842–0.994). The validation set had a cutoff of 0.423, a sensitivity of 0.844 (95% CI: 0.718–0.970), and a specificity of 0.911 (95% CI: 0.836–0.985) (Figure 2B). Based on this, calibration curves were plotted for the training and validation sets. The differences were not

statistically significant (*P* = 0.66, *P* = 0.79) according to the Hosmer-Lemeshow test (Figures 2C,D). It can be assumed that the fitted model is essentially free of deviation from the actual model. To evaluate the stability of the model, we performed 1,000 internal validations using the Bootstrap resampling method. The results showed that the mean absolute error (MAE) for the training and validation sets were 0.032 and 0.045, respectively, indicating good stability of the model across multiple resampled datasets. The clinical net benefit of the model was assessed using the decision curve analysis (DCA) curves (Figures 2E,F), showing clinical net benefits within risk thresholds of 0–0.985 and 0–0.96 for the training and validation sets, respectively.

## Discussion

Improving the diagnostic rate of AD is a challenge, as patients present with different symptoms depending on the affected area. When the dissection involves the coronary arteries, it can mimic acute coronary syndrome; when it involves the brachiocephalic or left common carotid artery, it can resemble an acute cerebrovascular event; and when the celiac trunk is affected, it can mimic an acute abdomen. Additionally, there have been case reports of more rare manifestations, such as painless paraplegia and isolated pharyngeal pain (9, 10). Therefore, it is necessary to analyze the symptoms of patients at their first admission, which can help us identify factors that may affect diagnosis. Schattner et al. (11) found that painless, atypical pain and fever are the three most common factors leading to the misdiagnosis of AD. Hirata et al. (12) found that walk-in presentations were the only predictor of misdiagnosis for acute type A AD in community hospitals. Our findings are similar to previous studies, indicating that the absence of typical back pain, non-acute onset of symptoms, and walk-in presentation are strong factors contributing to the misdiagnosis of AD. In our study, all 8 patients who initially presented with fever were misdiagnosed, and the cause of the fever may be related to underlying inflammation (13).Therefore, for patients with unexplained fever, AD should be considered as a potential cause. However, due to the small number of cases, our model did not ultimately include fever as a factor. Few previous studies have considered the duration of symptoms as a potential influence on misdiagnosis, but in our review of 219 cases, we found that symptom duration was the strongest influencing factor for misdiagnosis—the longer the symptoms persisted, the higher the probability of misdiagnosis. These factors ultimately lead the initial physicians to lower their vigilance, failing to consider AD.

Although multislice spiral CT angiography (CTA) is recognized as the gold standard for diagnosing AD, excessive use of CTA does not reduce the overall probability of misdiagnosis. Erdinç's study demonstrated that even in patients with typical tear-like pain symptoms, the aortic coarctation detection rate by CTA was only 5%. Therefore, after clinical evaluation, it is recommended to prioritize transthoracic echocardiography (TTE) and ultrasonography (USG) for the initial evaluation of suspicious cases (14). Ultrasound and CT are considered the final steps before performing diagnostic CTA in misdiagnosed patients. In our study, 67 of the 86 misdiagnosed patients (78%) were corrected through ultrasound and CT. According to Baliga et al. (15), the overall sensitivity of transthoracic echocardiography (TTE) for diagnosing AD is only 59%–83%, with a specificity of 63%–93%, while transesophageal echocardiography (TEE) has a sensitivity of up to 99% and a specificity of 89%. However, TEE requires experienced operators, and both modalities are limited in evaluating abdominal dissections. CT has an accuracy rate of over 90% in diagnosing AD and helps to rule out other diseases, but it cannot be performed at the bedside for critically ill patients. In our study, TEE was not performed on the patients, and the positive rates of both TEE and CT were lower than in previous studies (56.2% and 79.9%, respectively). First, differences in equipment and the skills of imaging specialists may affect the positive rates. A study on emergency CT misdiagnosis of AD found that in a review of 88 consecutive cases of AD, 13 cases (15%) were not identified by the reporting radiologists, who did not have a cardiovascular specialization (16). Second, our study included imaging examinations of both the thoracic and abdominal regions and some patients only completed one of these. If the dissection was located in the other region, it would lead to a negative result. Despite having more advanced diagnostic methods than before, the misdiagnosis rate of AD has not decreased (17). Therefore, even if patients do not exhibit typical symptoms, signs, or positive ultrasound or CT results, AD should not be ruled out.

Due to the intimal tear caused by AD, a large amount of tissue factor is released into the bloodstream, activating the coagulation system and forming a false lumen thrombus. This triggers a coagulation cascade reaction and activates the fibrinolytic system, leading to elevated plasma D-dimer levels. Therefore, D-dimer is currently recognized as the most relevant marker for AD. Nazerian et al. (18) found through a prospective multicenter study that a low ADDRS score (a scoring system used to assess the probability of AD) combined with a negative D-dimer result can effectively rule out AD, with an exclusion efficiency of 49.9% and a failure rate of 0.32% (19). Our study found that lower D-dimer levels still cannot rule out AD and are a weak predictor of misdiagnosis. First, we did not score the patients using the ADDRS system. Second, D-dimer typically increases 6 h after the onset of the dissection and is influenced by age, while the serum samples we collected were taken at the time of the patient's first admission. If the dissection occurred less than 6 h before sample collection, this could lead to a false-negative result (20). Therefore, lower D-dimer levels at early admission may affect clinical decision-making. Establishing an optimal age-adjusted D-dimer threshold based on different onset times may help clinicians identify AD early. Fibrinogen, as the precursor of D-dimer, abnormally increases when vascular endothelial cells are damaged. It acts as a direct coagulation factor involved in both intrinsic and extrinsic coagulation pathways and serves as a marker of the acute-phase response, indicating inflammation and injury (21). Li et al. (22) found that fibrinogen levels were significantly higher in AD patients than in the healthy population and were an independent risk factor for AD, especially in Stanford type B dissection patients. Our findings indicate that lower fibrinogen levels are a predictor of misdiagnosis, with a predictive strength even higher than that of D-dimer. Although the median fibrinogen level in the misdiagnosed group was higher than in the diagnosed group, this contradictory result may be due to the influence of the timing of AD onset or other confounding factors, suggesting that multiple factors should be considered in clinical diagnosis to improve accuracy. AD is an inflammatory disease, and previous studies have shown an increase in white blood cell count following the onset of dissection (23). Morello et al. (24) demonstrated that a white blood cell count >9*10^9^/L and platelet count <200*10^9^/L may increase the confidence of clinicians in diagnosing AD. Similarly, our study found that normal or low white blood cell counts are predictive of misdiagnosis, as the absence of an inflammatory response may mislead clinicians. However, we did not find a correlation between platelet count and misdiagnosis. While platelet count was significant in univariate analysis, it was not included in the final model, possibly because changes in platelets during AD are relatively indirect, leading to a lack of significant independent contribution. Currently, no specific serological diagnostic markers for AD have been identified. Wang et al. (25) found that soluble ST2 (sST2) demonstrated good overall diagnostic performance in suspected AD patients. However, the results of a European prospective study on this marker were not ideal, possibly due to differences in ethnicity (26). Lu et al. (27) demonstrated an association between AD and iron metabolism and developed the FLUTHE model using six serum markers, including serum iron and transferrin, to differentiate between AD and coronary artery disease. However, this model only showed good performance in patients with chest pain lasting more than 72 h. Additionally, studies on ceruloplasmin, serum amyloid A, and smooth muscle 22α as biomarkers are underway, and more effective diagnostic markers for AD may be discovered in the future.

A key finding of this study is that the time of symptom onset is the strongest influencing factor for the misdiagnosis of AD. The patient's symptoms and mode of admission also have good predictive power, while serological markers influence the misdiagnosis, though their predictive strength is weaker. Our model has certain advantages in predicting the risk of AD misdiagnosis, as all the indicators can be obtained through routine questioning and simple serological tests. Based on the scores corresponding to these indicators, clinicians can quickly and easily assess the risk of misdiagnosis and proceed with timely further examinations for high-risk patients to confirm or rule out AD. However, since our data comes from a single center, its representativeness may be limited. Additionally, the choice of variables in the model may have been influenced by the data collection process, potentially omitting other influential factors, which could affect the model's accuracy when applied to other centers. Therefore, its limitations must be considered in practical use, and decisions should be made in conjunction with clinical experience and other diagnostic tools.

## Limitation

First, this study is a single-center study, and the sample may not be representative of a broader population. Additionally, we did not assess the impact of the initial physicians on misdiagnosis outcomes. In fact, the experience level of the initial physicians may be directly related to the misdiagnosis, which means that our findings may not be widely applicable. Secondly, as a retrospective study, the quality of the data is influenced by the accuracy of previous case records, making it impossible to completely avoid selection bias and information bias.

## Conclusion

The time of symptom onset significantly influenced the misdiagnosis of AD, along with the lack of typical back pain, gradual symptom onset, and walk-in clinic visits. Conversely, lower levels of D-dimer, fibrinogen, and white blood cell counts had a less pronounced impact on misdiagnosis.

## Data Availability

The raw data supporting the conclusions of this article will be made available by the authors, without undue reservation.
